# Tumor promotion by γ and suppression by β non-muscle actin isoforms

**DOI:** 10.18632/oncotarget.3989

**Published:** 2015-05-04

**Authors:** Vera Dugina, Natalya Khromova, Vera Rybko, Oleg Blizniukov, Galina Shagieva, Christine Chaponnier, Boris Kopnin, Pavel Kopnin

**Affiliations:** ^1^ Belozersky Institute of Physico-Chemical Biology, Lomonosov Moscow State University, Moscow, Russia; ^2^ Blokhin Russian Cancer Research Center, Moscow, Russia; ^3^ Department of Pathology and Immunology, Faculty of Medicine, University of Geneva, CMU, Geneva, Switzerland

**Keywords:** cancer, actin isoforms, ERK1/2, PP1, p34-Arc, WAVE, cofilin1

## Abstract

Here we have shown that β-cytoplasmic actin acts as a tumor suppressor, inhibiting cell growth and invasion *in vitro* and tumor growth *in vivo*. In contrast, γ-cytoplasmic actin increases the oncogenic potential via ERK1/2, p34-Arc, WAVE2, cofilin1, PP1 and other regulatory proteins. There is a positive feedback loop between γ-actin expression and ERK1/2 activation. We conclude that non-muscle actin isoforms should not be considered as merely housekeeping proteins and the β/γ-actins ratio can be used as an oncogenic marker at least for lung and colon carcinomas. Agents that increase β- and/or decrease γ-actin expression may be useful for anticancer therapy.

## INTRODUCTION

At the moment six highly conserved actin isoforms in vertebrates are known: four muscle and two non-muscle. The main actin isoform studied in the context of carcinogenesis is the α-smooth muscle actin (*ACTA2*), which is expressed in normal smooth muscle cells, in myoepithelial cells and in myofibroblasts including tumor-associated fibroblasts [1]. Muscle actins are mainly tissue specific, whereas non-muscle β- and γ-cytoplasmic actins (β- and γ-actins hereafter), encoded by *ACTB* and *ACTG1* genes respectively, are ubiquitously expressed in almost all cells [2, 3] and can be essential for cell survival [4]. The proportion of β- and γ-actins depends on the cell type [2, 5-7]. Their expression differs in various tissues, depending not only on differentiation, but on functional activity of the cell. Actin isoforms expression levels are often associated with different pathological processes [8]. Non-muscle actin isoforms play crucial roles in cell migration, division and even intracellular signaling [9]. The purpose of this study was to explore the roles of non-muscle cytoplasmiс β- and γ-actin isoforms expression changes in cell transformation and tumor progression. These proteins differ only in four amino acids near the N-terminus [2] and are expressed in normal epithelial cells. Previously, we have shown that β-actin is connected with contraction and adhesion, whereas γ-actin predominantly forms the cortical network necessary for shape flexibility and motile activity of normal fibroblasts and epithelial cells [10].

The majority of studies consider actins to play only an architectonic role. Despite the mechanisms of actin-dependent migration have been deeply investigated, less is known about possible specific functions of the cytoplasmic actin isoforms in this process. Cell motility can depend on non-muscle β- and γ-actins during embryogenesis and in normal human subcutaneous fibroblasts, with γ-actin determining the directionality of cell movement [10, 11]. Partial RNAi suppression of γ-actin expression in SH-EP neuroblastoma cells resulted in a significant decrease in wound healing and transwell migration. Similarly, the knockdown of γ-actin significantly reduced speed of motility and severely affected the cell's ability to explore, which was, in part, due to a loss of cell polarity [12]. Some data on γ-actin regulation of cell migration and ROCK signaling has also been obtained [12, 13]. Recent data on cytoplasmic actins as AcGFP fusion proteins overexpressed in colon adenocarcinoma suggest that both actin isoforms have an impact on cancer cell motility, with the subtle predominance of γ-actin [14].

We have previously shown that the relative level of β-actin was decreased in tumors compared with corresponding normal tissue (cervical, breast) while γ-actin was expressed evenly and diffuse in all studied normal and malignant tissues [15-17].

The aim of this work was to study the occurrence of the above mentioned actins expression changes in various types of common cancers such as colon and lung. And, most importantly, we aimed to study the role of β- and γ-actins in cell transformation and/or tumor progression as well as to find some proteins through which of actin isoforms could influence these processes.

## RESULTS

### Cytoplasmic actins expression differs in normal and carcinoma cells of human lung and colon

**Figure 1:**
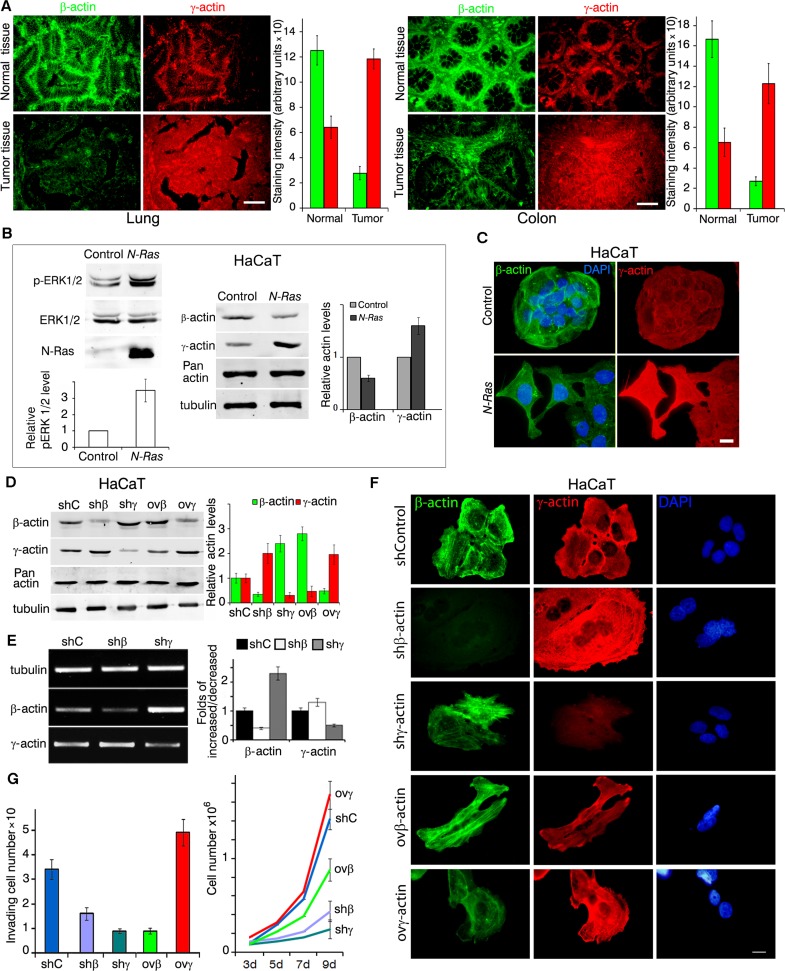
Expression of cytoplasmic actins in normal and transformed cells (A-C); expression of cytoplasmic actins and HaCaT cellular characteristics (D-G) **A.** Immunofluorescent staining for β-actin (green) and γ-actin (red) of representative pairs of NSCLC and Colon Cancer samples with matching normal tissue samples. Scale bars represent 50μm. Graphs represent staining intensity of biopsies (Mean ± SD). **B.** WB analysis of HaCaT cells with exogenous expression of active *N-RasD*^13^. Graphs represent relative actins expression (Mean ± SD). **C.** Immunofluorescent staining for β-actin (green) and γ-actin (red) of *N-RasD*^13^ transformed HaCaT cells. Scale bars represent 10 μm. **D.** WB analysis of HaCaT cells with exogenous expression of β- or γ-actins and corresponding shRNAs. shC is sh to GFP. Graphs represent relative actins expression (Mean ± SD). **E.** Semi-quantitative RT-PCR analysis of β- or γ-actins expression in cells with corresponding shRNAs. Graphs represent folds of RNA expression in comparison to control cells (Mean ± SD). **F.** Immunofluorescent staining of HaCaT cells with altered β- or γ-actins expression. Scale bars represent 10 μm. **G.** HaCaT cells with silenced or exogenously expressed non-muscle cytoplasmic actins that crossed the matrigel-coated membranes (left). Graphs represent mean ± SD. HaCaT proliferation dynamics with exogenous expression or silenced of β- or γ-actins (right). Error bars are SD.

We have studied the distribution of β- and γ-actins in normal cells compared with malignant human lung and colon epithelial cells. First, we studied β- and γ-actin expression in matching pairs of neoplastic and normal tissues (20 non-small cell lung cancer (NSCLC) and 15 colon cancer). Significant decrease of β-actin staining was observed in NSCLC compared with non-malignant tissue. Quantification of relative fluorescent signal revealed 4-times lower intensity of β-actin staining in cancer lesions compared with matching normal tissues. γ-Actin staining was doubled in carcinoma compared with normal tissues (Figure 1A, lung). Similar results were obtained for colon cancer (Figure 1A, colon): 5 times lower intensity of β-actin and about double enhancement of γ-actin staining in neoplastic *vs* normal cells.

### Ras-transformation reduces β-actin and stimulates expression of γ-actin

Activation of the Ras pathway is one of the most frequent molecular abnormalities of various malignancies, including lung and colon cancers. We introduced *N-RasD^13^* [18] into human normal spontaneously immortalized keratinocytes HaCaT. Exogenous *N-Ras*-expressing cells developed a transformed fibroblast-like phenotype that can be described as EMT III type [19, 20]. Control epithelial cells had well-developed β-actin circular bundles connected with intercellular contacts (Figure 1C). Ras-induced ERK1/2 activation was accompanied with a significant β-actin decrease and γ-actin increase while the total actin amount remained unchanged (Figure 1B, Sup. Figure 1).

### Non-muscle actin isoforms in phenotype of normal HaCat cells

We created stable HaCaT derivatives with overexpressed or silenced β- or γ- actins to check their presumable transforming potency (Figure 1D, 1E). Down-regulation of β-actin by shRNA or γ-actin overexpression induced a spread and fan-shaped phenotype. Down-regulation of γ-actin or β-actin overexpression induced a more contractile phenotype (Figure 1F). The total amount of cytoplasmic actin remained unchanged. Any isoform expression alteration led to the opposite effect on the other isoform both on protein (Figure 1D) and on mRNA levels (Figure 1E).

Changes of actins expression can induce phenotypical traits of transformed cells as well as influence their physiological properties. Basal weak invasion of HaCaT cells in matrigel assay increased significantly upon γ-actin overexpression (Figure 1G, left). On the other hand, β-actin overexpression significantly reduced HaCaT proliferation in culture. shRNA β- or γ-actin down-regulation reduced proliferation (Figure 1G, right), while γ-actin overexpression stimulated it. Despite its pro-invasive and pro-proliferative effect in immortalized keratinocytes, γ-actin overexpression was not sufficient to induce HaCaT tumorigenicity in a xenograft model. So to study the potential γ-actin oncogenesis-promoting role we further used lung adenocarcinoma A549 and colon carcinoma HCT116 cell lines as well as their xenografts.

### Actin isoforms down- and up-regulation alter cancer cells phenotype

**Figure 2:**
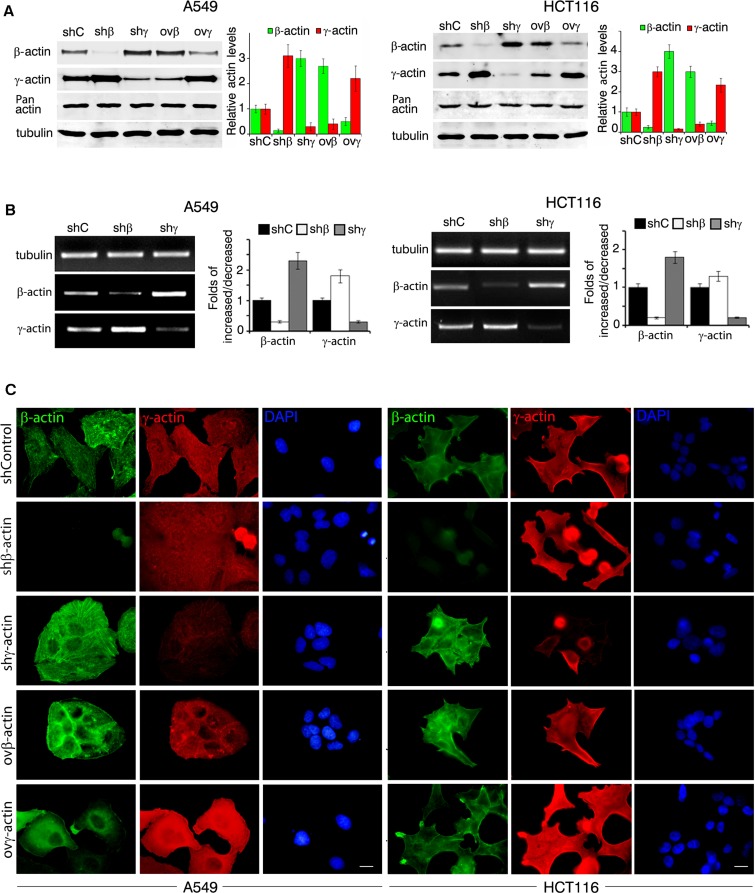
β- and γ-actins in neoplastic cells phenotype **A.** WB analysis of A549 cells (left panel) and HCT116 cells (right panel) with exogenous expression of β- or γ-actins and corresponding shRNAs. Graphs represent relative actins expression (Mean ± SD). **B.** Semi-quantitative RT-PCR analysis of β- or γ-actins expression in A549 (left panel) and HCT116 (right panel) with corresponding shRNAs. Graphs represent folds of RNA expression in comparison to control cells (Mean ± SD). **C.** Immunofluorescent staining of A549 cells (left panel) and HCT116 cells (right panel) with exogenous expression of β- or γ-actins and corresponding shRNAs. Scale bars represent 10 μm.

We obtained A549 (Figure 2, left) and HCT116 (Figure 2, right) derivatives with overexpressed or silenced β- and γ-actins. shRNAs depletions were specific for each of cytoplasmic actin isoforms (Figure 2A). β-Actin depletion in A549 and HCT116 as in HaCaT cells was accompanied by γ-actin up-regulation. γ-Actin down-regulation resulted in β-actin enhancement. The compensatory mechanism for cytoplasmic actin isoforms expression acts both on mRNA and protein levels (Figure 2A, 2B).

Parental A549 and HCT116 cultures have moderate or low levels of β-actin staining, mainly presented by diffuse or disorganized β-actin bundles and evenly moderate level of γ-actin cortical staining. In A549 (Figure 2C, left) and HCT116 (Figure 2C, right) cells further shRNA β-actin down-regulation induced a spread and motile cell phenotype and γ-actin down-regulation induced a more contractile one. β-Actin overexpression inhibited spread and motile phenotype of both cell lines, which lead to a “more normal” epithelial phenotype. γ-Actin overexpression induced a “more transformed” fan-shaped scattered phenotype. This demonstrates that γ-actin predominance contributes to aggressive carcinoma cells phenotype.

### Growth kinetics in cell culture and xenograft nude mouse model: γ-actin stimulates carcinoma cells proliferation *in vitro* and *in vivo*

**Figure 3:**
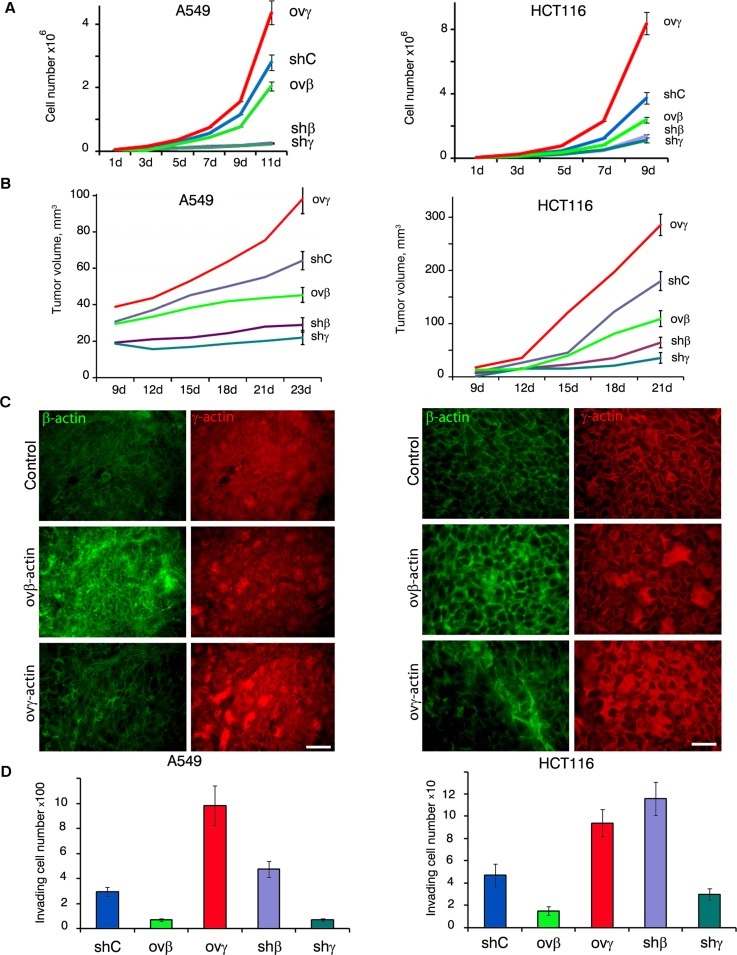
β- and γ-actins in malignant growth **A.** Proliferation dynamics of A549 (left panel) and HCT116 (right panel) cells with exogenous expression or silenced of β- or γ-actins. Error bars are SD. **B.** Dynamics of xenographt growth after subcutaneous injection (10 mice per group) of A549 (left panel) and HCT116 (right panel) cells with exogenous expression or silenced of β- or γ-actins. Error bars are SD. **C.** Immunofluorescent staining for β-actin (green) and γ-actin (red) of tumors xenographts after 23 days of subcutaneous growth. Scale bars represent 50 μm. **D.** Invasion of A549 (left panel) and HCT116 cells through matrigel-coated membranes (Mean ± SD).

Overexpression of γ-actin in A549 and HCT116 cells led to a significant acceleration of proliferation. β-Actin exogenous expression slightly slowed down cell division while silencing of studied actins led to significant alterations in proliferation *in vitro* (Figure 3A). The latter phenomenon could be explained by impaired cytokinesis in β-actin-depleted cells [10] and by the necessity of both actin isoforms for mitosis. Full inactivation β- or γ-actins inhibits cells division. Subcutaneous A549 and HCT116 tumors with overexpressed γ-actin grew faster compared with control cells. β-Actin overexpression slightly slowed down xenografts growth. Silencing of β- and γ-actins led to A549 and HCT116 subcutaneous growth complete arrest (Figure 3B; Sup. Figure 2). Overexpression of actin isoforms remained unchanged after 21 days of subcutaneous growth in both cell lines (Figure 3C).

### γ-actin overexpression stimulates invasion

In Boyden chambers assay mirroring cellular characteristics linked to malignant features cells with down-regulated γ-actin demonstrated lower invasiveness *in vitro* compared with controls. Similar to γ-actin-deficient cell cultures, invasion was almost blocked in β-actin overexpressing A549 and HCT116. On the contrary, γ-actin stimulated invasion of both cell lines; HCT116 cells with down-regulated β-actin also invaded more effectively (Figure 3D).

### Reciprocal regulation between β- and γ-actins and ERK1/2

**Figure 4:**
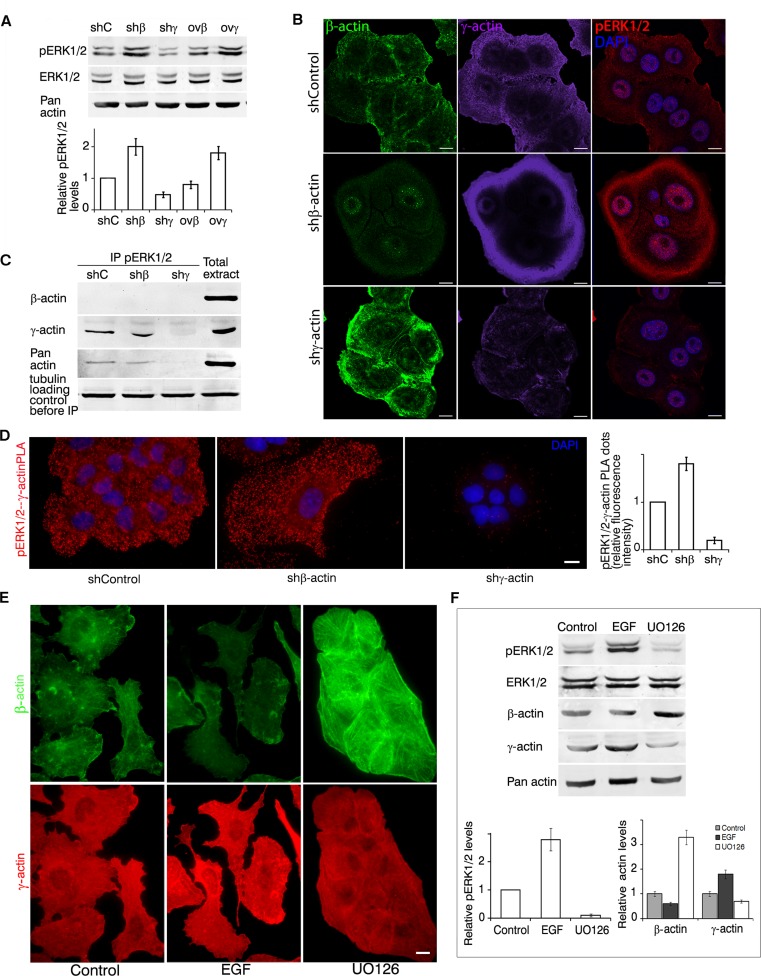
γ-actin and ERK1/2 are mutually regulated **A.** WB analysis of A549 cells with exogenous expression of β- or γ-actins and corresponding shRNAs. Graphs represent relative pERK1/2 expression (Mean ± SD). **B.** LSM of A549 cells with down-regulated β- or γ-actins with β-actin (green), γ-actin (purple) or pERK1/2 (red) immunofluorescent staining. Scale bars represent 10 μm. **C.** pERK1/2 immunoprecipitation analysis of A549 cells with down-regulated β- or γ-actins. **D.** pERK1/2/γ-actin PLA analysis of A549 cells with down-regulated β- or γ-actins. Scale bar represents 10 μm. Graph represents relative fluorescence intensity (Mean ± SD). **E.** LSM of A549 cells treated with EGF or UO126 with β-actin (green) or γ-actin (red) immunofluorescent staining. Scale bars represent 10 μm. **F.** WB analysis of A549 cells treated with EGF or UO126. Graphs represent relative pERK1/2 or β-/γ-actin levels (Mean ± SD).

Both cell proliferation and invasion *in vitro* are stimulated by growth factors activating the canonical MAPK pathway. We supposed that changing actin isoforms ratio could influence the intensity of this signaling. Indeed γ-actin predominance in A549 and HCT116 was associated with ERK1/2 activation (according to western blot analysis, Figure 4A).

Control A549 cells exhibited moderate levels of cytoplasmic phosphorylated ERK1/2 (pERK1/2) staining according to Laser Scanning Microscopy (LSM) (Figure 4B). Silencing of β-actin led not only to γ-actin increase, but also to cytoplasmic and nuclear phospho-ERK1/2 accumulation. LSM revealed co-localization of γ-actin and phospho-ERK1/2 especially at the leading edge of control and β-actin-deficient cells. γ-Actin overexpression led to similar results. β-Actin predominance diminished phospho-ERK1/2 staining (Sup. Figure 3).

We confirmed phospho-ERK1/2 and γ-actin binding by co-immunoprecipitation (Figure 4C). For these experiments we used A549 cells with silenced β- or γ-actins to minimize the influence of actin isoforms on biochemical data. Using PLA (Proximity Ligation Assay) [21, 22] we verified the pERK1/2−γ-actin co-localization (Figure 4D). PLA using γ-actin and pERK1/2 demonstrated highly specific and strong signals as multiple cytoplasmic dots in control and β-actin-deficient cells (Figure 4D). Comparative fluorescent signals of pERK1/2−γ-actin PLA dots in control and actins-depleted A549 cells quantification is shown in Figure 4D. β-Actin and pERK1/2 antibodies gave fluorescent signals on the level of background.

EGF treatment of A549 cells induced fast and stable ERK1/2 phosphorylation, while UO126 (a selective MEK inhibitor) completely blocked activation of these MAP kinases (Figure 4E, 4F). EGF led to γ-actin predominance and disappearance of β-actin bundles in cells, while UO126 stimulated β-actin bundles formation (Figure 4E, 4F).

So these experiments showed for the first time that active ERK1/2 interacts with γ-actin in carcinoma cells. Moreover, ERK1/2 activation leads not only to stimulation of cell proliferation and morphological changes in intercellular architecture, but also to β-actin down- and γ-actin up-regulation. So we assume that alterations in β/γ-actin ratio may partly explain the phenotypical changes upon ERK1/2 activation.

### γ-actin selectively interacts with Arp2/3 complex

Actin polymerization at the leading edge is crucial for any type of cell migration [23]. We have previously demonstrated that both β- and γ-actins are enriched in protrusions with active actin branching and nucleation at the leading edge of normal subcutaneous fibroblasts [10]. It has been previously shown that ERK-MAPK signaling drives lamellipodial protrusions with WAVE2 regulatory complex activation [24]. ERK pathway activation during migration leads to actin reorganization by WAVE2/Arp2/3 polymerization complex [25-27]. Our data on spreading fibroblasts with specific N-WASP inhibitor [10] and preliminary experiments on carcinoma cells using Arp2/3 inhibitor CK666 (not shown) allowed us to hypothesize diverse polymerizing systems/pathways for β- and γ-actins. As ERK1/2 activation is associated with γ-actin at the leading edge we assumed that Arp2/3 polymerization complex could predominantly interact with γ-actin during actin branching and migration.

**Figure 5:**
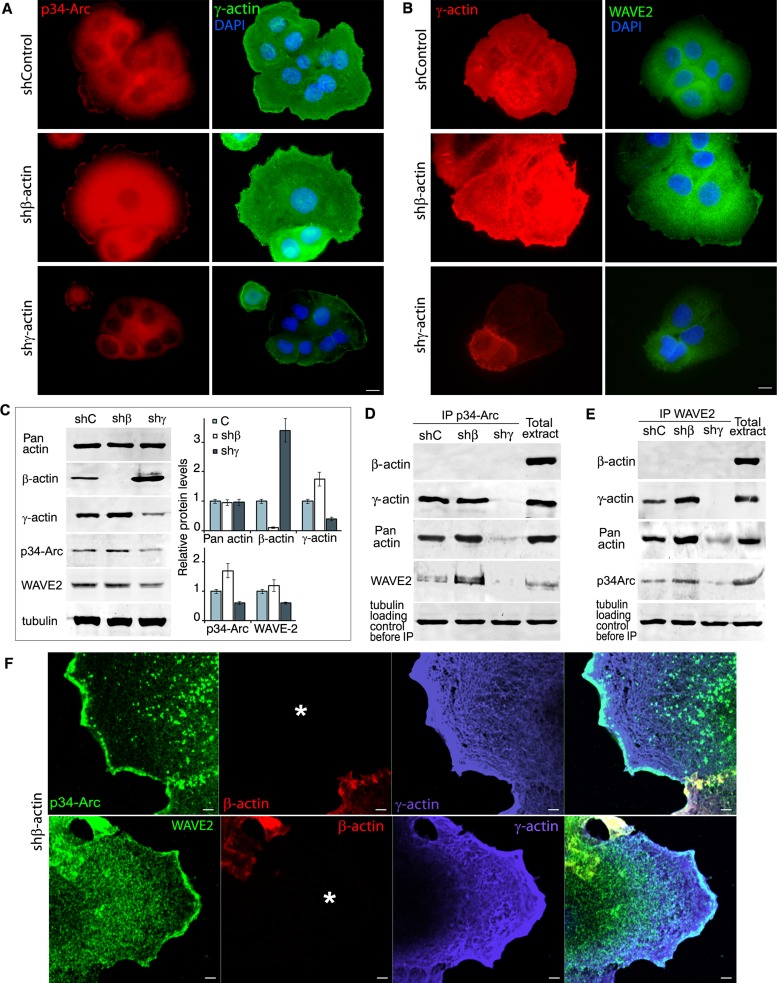
γ-actin co-localizes with p34-Arc and WAVE2 and regulates them **A.** p34-Arc (red) or γ-actin (green) immunofluorescence images of A549 cells with down-regulated β- or γ-actins. Scale bars represent 10 μm. **B.** γ-Actin (red) or WAVE2 (green) immunofluorescence images of A549 cells with down-regulated β- or γ-actins. Scale bars represent 10 μm. **C.** WB analysis of A549 cells with down-regulated β- or γ-actins. Graphs represent relative protein levels (Mean ± SD). **D.** p34-Arc immunoprecipitation analysis of A549 cells with down-regulated β- or γ-actins. **E.** WAVE2 immunoprecipitation analysis of A549 cells with down-regulated β- or γ-actins. **F.** LSM of A549 cells with down-regulated β-actin with p34-Arc (green, upper panel), WAVE2 (green, lower panel), β-actin (red) or γ-actin (purple) immunofluorescent staining. Scale bars represent 2μm. Asterisk marks the cell with silenced β-actin.

Immunofluorescence microscopy of A549 cells showed that β-actin silencing induced enhancement of p34-Arc (member of the Arp2/3 polymerization complex) and of WAVE2 staining (especially at the leading edge), while γ-actin down-regulation reduced it (Figure 5A, 5B). Western blot analysis (Figure 5C) revealed that γ-actin predominance induced by β-actin down-regulation moderately stimulated p34-Arc expression. γ-Actin depletion reduced p34-Arc and WAVE2 expression.

Using Co-IP, both γ-actin and WAVE2 were detected together with p34-Arc (Figure 5D) and both γ-actin and p34-Arc were detected together with WAVE2 (Figure 5E). Confocal LSM of β-actin-deficient A549 cells also revealed co-localization of both p34-Arc and WAVE2 with well-developed γ-actin network, especially in lamellipodia (Figure 5F). Using PLA with γ-actin and p34-Arc or WAVE2 antibodies we obtained specific and strong signals as multiple cytoplasmic dots at the leading edge of control and, more obviously, of β-actin-deficient cells (Sup. Figure 4A). Comparative quantification of pERK1/2−γ-actin PLA dots in control and actins-depleted A549 cells is shown in Sup. Figure 4B. With β-actin/p34-Arc or β-actin/WAVE2 antibodies we got fluorescent signals on the level of background.

To conclude we have shown a direct γ-actin−WAVE2/Arp2/3 interaction at the leading edge of the cell and discovered that γ-actin and p34-Arc/WAVE2 expression levels correlate in the studied cells.

### Cofilin1 selectively interacts with γ-actin

Cofilin1 is a regulator of actin filaments reorganization in Arp2/3-dependent actin branching at the leading edge [28-32] and cofilini1 is important for cancer cells motility [33-37].

**Figure 6:**
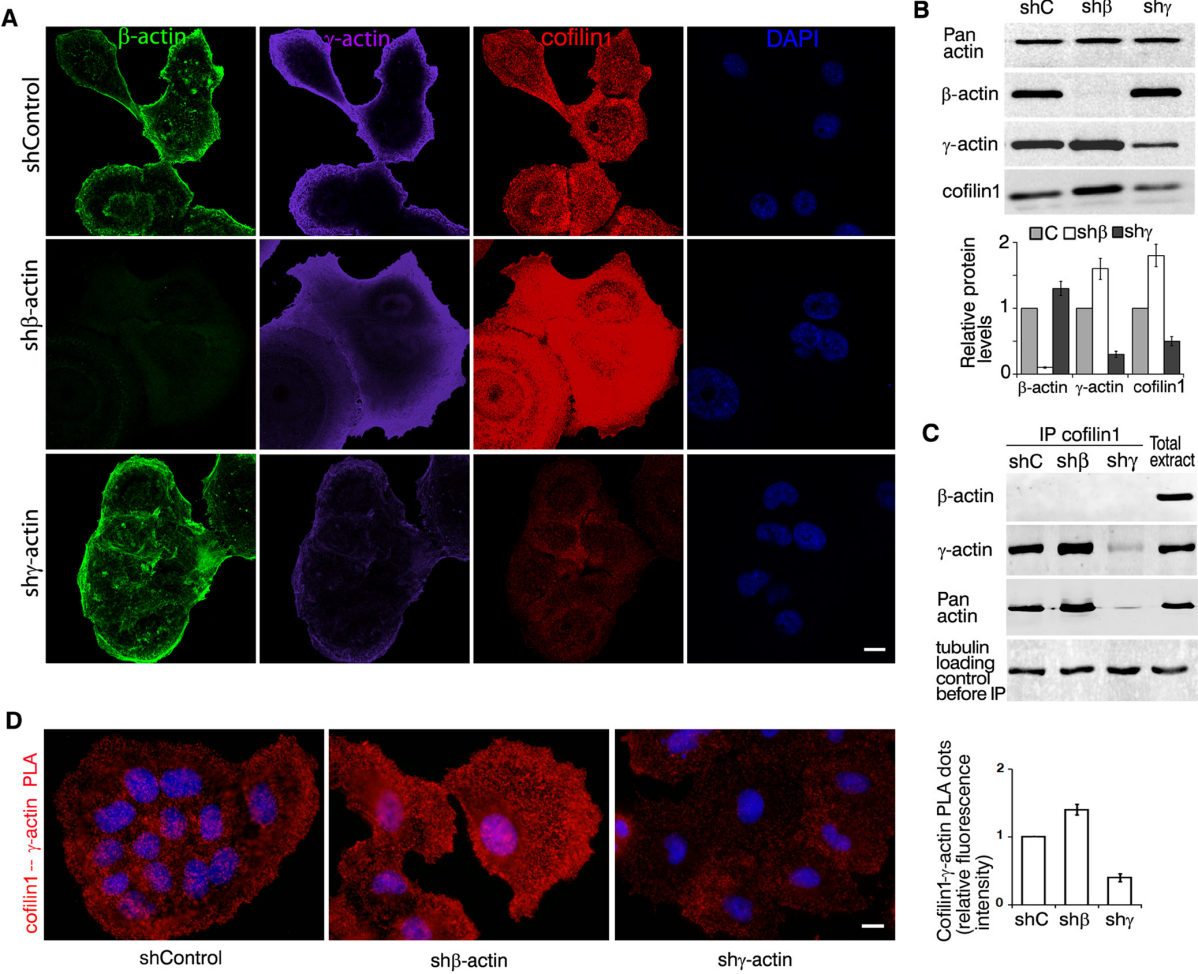
γ-actin co-localizes with cofilin1 and regulates it **A.** LSM of A549 cells with down-regulated β- or γ-actins with β-actin (green), γ-actin (purple), cofilin1 (red) or DAPI (blue) immunofluorescent staining. Scale bars represent 10 μm. **B.** WB analysis of A549 cells with down-regulated β- or γ-actins. Graphs represent relative protein levels (Mean ± SD). **C.** Cofilin1 immunoprecipitation analysis of A549 cells with down-regulated β- or γ-actins. **D.** Cofilin1/γ-actin PLA analysis of A549 cells with down-regulated β- or γ-actins. Scale bar represents 10 μm. Graph represents relative fluorescence intensity (Mean ± SD).

Using LSM of cofilin1 in A549 cells with silenced β- or γ-actins (Figure 6A) we have shown that γ-actin predominance induces enhancement of cofilin1 staining (especially at the leading edge). Co-localization of cofilin1 with γ-actin (especially in β-actin-depleted cells) was demonstrated (Sup. Figure 5). Selective silencing of actin isoforms led to modulations of cofilin1 expression that correlated with γ-actin expression (Figure 6B). IP experiments confirmed cofilin1−γ-actin interaction (Figure 6C). γ-Actin/cofilin1 PLA demonstrated specific and strong signals at the leading edge of control and of β-actin-deficient cells (Figure 6D). β-Actin/cofilin1 PLA gave fluorescent signals on the level of background.

### PP1-regulation

Signaling pathways activation and deactivation is regulated through phosphorylation by kinases and dephosphorylation by phosphatases. Existence of different phosphatases in various complexes contributes to diversity of their effects [38].

**Figure 7:**
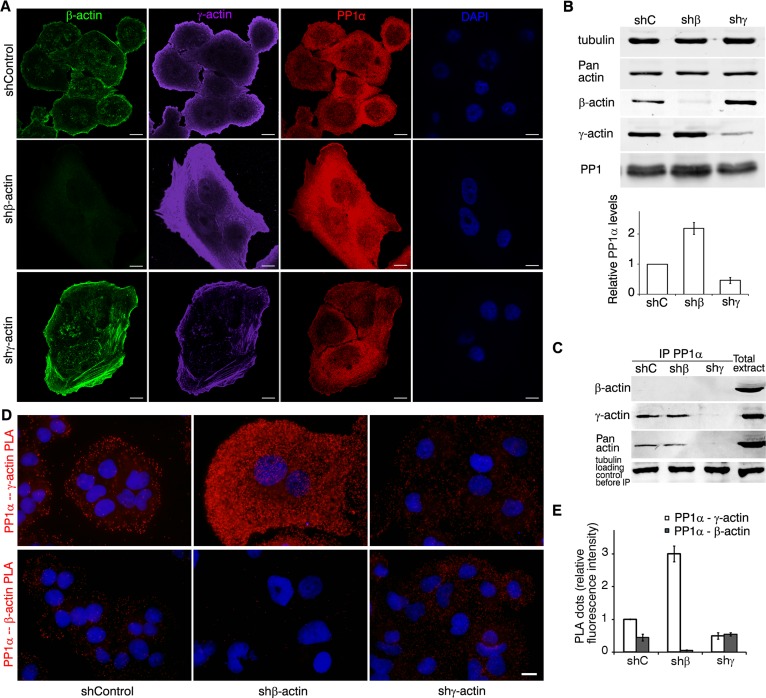
γ-actin co-localizes with PP1 and regulates it **A.** LSM of A549 cells with down-regulated β- or γ-actins with β-actin (green), γ-actin (purple), PP1α (red) or DAPI (blue) immunofluorescent staining. Scale bars represent 10 μm. **B.** WB analysis of A549 cells with down-regulated β- or γ-actins. Graphs represent relative PP1α levels (Mean ± SD). **C.** PP1 immunoprecipitation analysis of A549 cells with down-regulated β- or γ-actins. **D.** PP1α/γ-actin (upper panel) and PP1α/β-actin PLA analysis of A549 cells with down-regulated β- or γ-actins. Scale bar represents 10 μm. **E.** Comparative quantification of PP1α−β-/γ-actin PLA dots. Graph represents relative fluorescence intensity (Mean ± SD).

A preliminary screening has revealed that Protein Phosphatase 1 (PP1) is involved in γ-actin-dependent signaling. PP1 is one of the main dephosphorylating enzymes. In particular, PP1 can dephosphorylate cofilin1 and thus activate it [39]. PP1α confocal LSM of control A549 cells showed cytoplasmic staining. β-Actin down-regulation in A549 induced a moderate enhancement of PP1α cytoplasmic staining (Figure 7A). PP1α co-localized with γ-actin at the cellular leading edge. Western blot analysis (Figure 7B) confirmed reduction of PP1α expression as a result of γ-actin down-regulating. Co-IP showed γ-actin (not β-actin) binding with PP1α (Figure 7C).

γ-Actin−PP1α PLA demonstrated specific and strong signals in the cytoplasm of control and, more obviously, of β-actin-deficient cells. β-Actin−PP1α PLA demonstrated low, but significant fluorescence in control and in γ-actin-deficient cells (Figure 7D, 7E).

## DISCUSSION

Until recently non-muscle cytoplasmic β- and γ-actins were considered only to play structural roles in cellular architecture and motility. They were viewed as products of housekeeping genes and β-actin was commonly used as internal control in various biochemical experiments. The difference between β- and γ-actins was poorly studied because of lack of specific antibodies distinguishing these two proteins. Embryonic lethality, with γ-cytoplasmic actin null mice (*Actg1*−/−) dead within 48h after birth [11], complicated the study of non-muscle actin isoforms. Selective siRNA-mediated knockdown of γ-cytoplasmic actin as compared to β-actin, induced epithelial-to-mesenchymal transition of various epithelial cells, which manifested in increased expression of contractile proteins along with inhibition of genes responsible for cell proliferation [40]. Some data indicated a role of β- and γ-actins in carcinogenesis: components of the Arp2/3 complex were up-regulated in colorectal cancers [41], increased *ACTG1* and reduced *ACTB* levels were identified in osteosarcoma analysis [42].

In our experiments we have observed that stable shRNA-mediated knockdown of γ-actin or enhancement of β-actin induces normalization of carcinoma cells phenotype. We have showed for the first time that β- and γ-actins have distinct roles in tumorigenesis. γ-Actin, as opposed to β-actin, is significantly increased in colon and lung carcinomas when compared with normal tissues. Oncogenic Ras enhances γ-actin expression and reduces β-actin in immortalized HaCaT cells. γ-Actin overexpression in these cells leads to significant phenotypic changes and to invasion induction reminiscent of Ras activation. These alterations are not sufficient to induce tumor growth of HaCaT cells in immuno-compromised mice. Nonetheless we have shown that the increase of γ-actin level is a basic characteristic of cell transformation and tumor progression.

We have also discovered the existence of a ratio between these two proteins. A shift in the amount of one isoform is compensated by a reciprocal change in another form that can be explained by transcriptional regulation, at least partially. This phenomenon needs further investigation. The relationship of quantity occurred both in normal and in neoplastic cell lines with total actin amount remaining unchanged upon of β- or γ-actin overexpression or silencing.

We have discovered that γ-actin overexpression *(i)* accelerates proliferation of carcinoma cells both *in vitro* and *in vivo* in subcutaneous xenographts and *(ii)* results in formation of locomotor phenotype and increased invasion *in vitro*. On the contrary, β-actin overexpression suppresses the above properties and leads to more epithelial phenotype/differentiation.

To investigate the mechanism of these phenomena we studied the role of individual actin isoforms alterations in ERK1/2 MAP-kinases stimulation of cell proliferation and migration [43-45]. We showed that γ-actin predominance led to ERK1/2 activation. We also discovered ERK1/2−γ-actin binding and, even more, up-regulation of γ-actin with down-regulation of β-actin upon ERK1/2 activation and vice versa. So we can conclude that there is a positive feedback mechanism between MAP-kinases activation and γ-actin amount, which enhances malignant traits of neoplastic cells. Taking into account that activation of ERK1/2 MAP-kinases in response to activation of various oncogenes is observed in a majority of human neoplasias, we can assume that γ-actin predominance may present a universal malignant feature.

We also discovered γ-actin selective binding to *(i)* Arp2/3 complex member p34-Arc, *(ii)* WAVE2, a cofactor essential for actin polymerization, and *(iii)* cofilin1 required for proper actin branching at the leading edge. It is worth to mention that cofilin1 displays the above properties in a dephosphorylated state and that one of its putative phosphatases, namely PP1, also binds to γ-actin. All these interplays take place at the leading edge of the cell, as registered by PLA. So we can assume that these interactions could serve as a basis for the structural explanation of γ-actin necessity for migration and invasion. Moreover, γ-actin with ERK1/2 complex could act as a scaffold for further “signalosome” assembly.

We discovered γ-actin dual role in tumorigenesis. Structurally it supports the leading edge formation. It also plays a signal-conductive role. γ-Actin predominance enhances not only the probability of interactions necessary for cellular movement but also the intensity of mitogenic signaling. Vice versa: oncogenic stimuli up-regulate γ-actin in a transformed cell.

**Figure 8:**
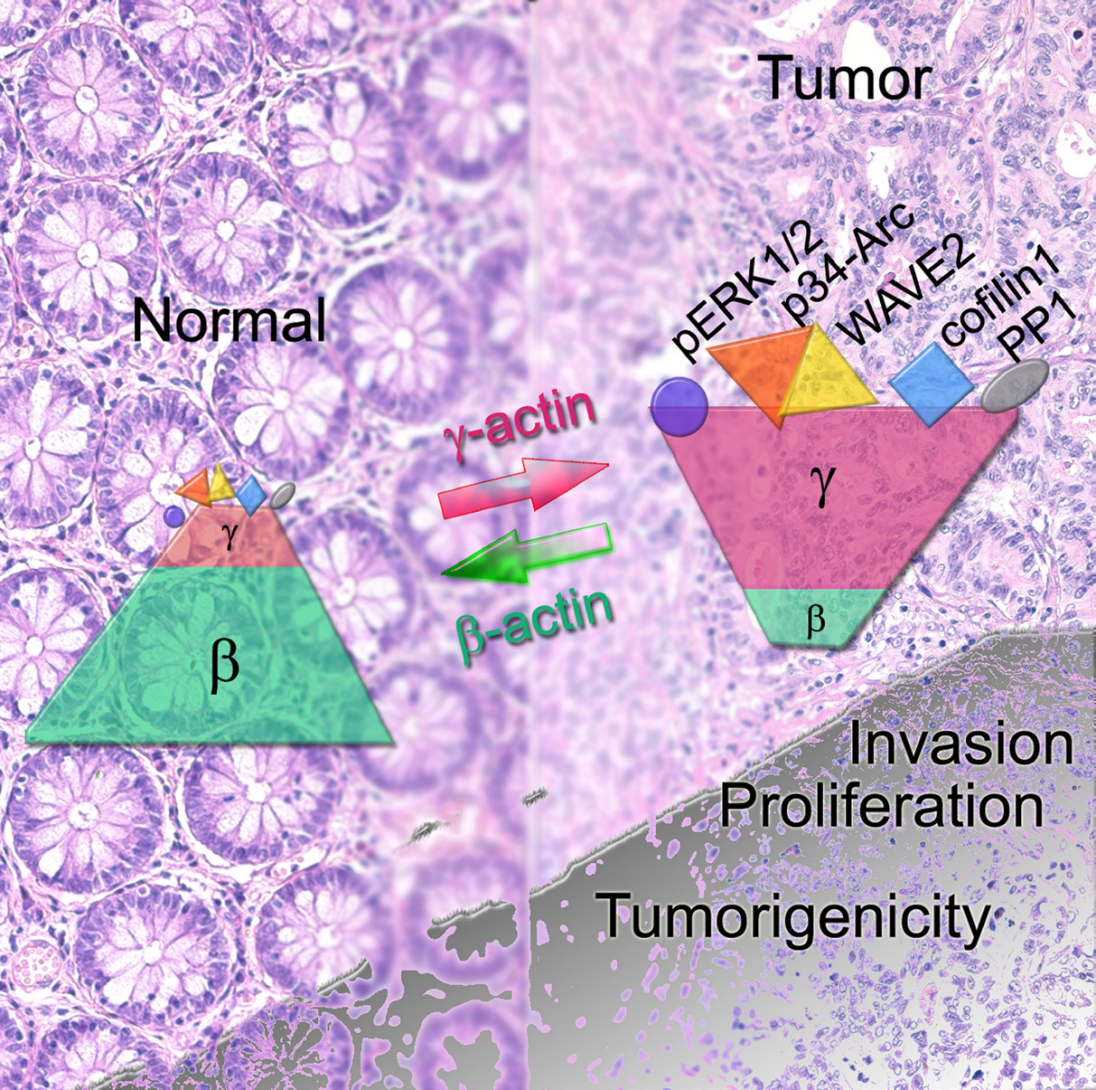
(SCHEME) In cancer cells the ratio of non-muscle cytoplasmic β- and γ-actins shifts towards γ-actin predominance. γ-Actin (unlike β-actin) can interact with both structural (components of Arp2/3 and WAVE2 complexes, cofilin1 that is dispensable for movement and cellular architecture) and signaling proteins (ERK1/2, PP1). Upon malignant transformation γ-actin becomes overexpressed. The cell acquires a more mesenchymal phenotype. It becomes more invasive and grows faster both *in vitro* and *in vivo*. β-Actin predominance in a transformed cell has an opposite effect: a more “normal” epithelial phenotype, impaired invasion and growth. γ-Actin could be considered as a weak oncogene and β-actin as an anti-oncogene.

To conclude, we have been the first to demonstrate the many-sided role of actin isoforms in cell behavior. The ability of γ-actin to bind key signaling molecules such as PP1α and ERKs and to modify their activity as well as to regulate actin network system *via* selective interaction with proteins of Arp2/3-complex, WAVE2 and cofilin1 allows to consider it an oncogene involved in tumorigenesis. This suggestion is strongly supported by γ-actin up-regulation in all studied samples of human lung and colorectal carcinomas along with a positive feedback loop with ERK1/2 activation. The results of our experiments are shown in Figure 8. We expect that restoring normal ratio of actins isoforms by diminishing γ-actin expression might provide a new route to improve anticancer therapy. New methods selectively targeting the actin cytoskeleton could be very perspective in this context [46, 47].

## MATERIALS AND METHODS

### Cells

Human colon carcinoma HCT116 cell line (ATCC # CCL-247), lung adenocarcinoma A549 cell line (ATCС # CCL-185) and immortalized keratinocytes HaCaT (CLS # 300493).

### DNA constructs

pBabe-puro with *N-RasD^13^* mutant was previously described [18]. For β- and γ-actins overexpression human total cDNA's obtained from normal human fibroblasts was used for reverse PCR (oligo dT and M-MLV RT (Promega)) with specific β- and γ-actin primers with BamHI/EcoRI adapters (forward BamHI-β-actin 5′-ATATGGATCCATGGATGATGATATCGCCGCG-3′; forward BamHI-γ-actin 5′-ATATGGATCCATGGAAGAAGAGATCGCCGCG-3′ and reverse EcoRI-β/γ-actin5′-ATATGAATTCCTAGAAGCATTTGCGGTGGACGAT-3′) and PfuUltraII (Stratagene) PCR products were cloned into the lentiviral pLenti6 vector (Invitrogen) with modified polylinker. Accuracy of PCR clones was verified by DNA sequencing.

siRNAs for β- and γ-actins specific hairpin structures containing 21-bp sequences were synthesized and cloned into pLKO.1-puro (Sigma-Aldrich): 5′-AATGAAGATCAAGATCATTGC-3′, 5′-CAAATATGAGATGCGTTGTTA-3′, 5′-TAGCATTGCTTTCGTGTAAAT-3′ (corresponding to β-actin mRNA ref|NM_001101.3| in 1056-1076; 1465-1475; 1575-1595 positions) and 5′-AAGAGATCGCCGCGCTGGTCA-3′, 5′-GGGCTGGCAAGAACCAGTTGTTT-3′, 5′-CAGCAACACGTCATTGTGTAA-3′ (corresponding to γ-actin mRNA ref|NM_001199954.1| in 266-286; 1790-1811; 2057-2077). All shRNAs constructs demonstrated similar biological effects, the most effective constructs (5′-CAAATATGAGATGCGTTGTTA-3′ for β-actin and 5′-CAGCAACACGTCATTGTGTAA-3′ for γ-actin) were chosen and the results described in the article. pLKO.1-shGFP-puro targeting eGFP (GenBank Accession No. pEGFP U55761) was used as a control. Oligonucleotides synthesis and DNA sequencing was performed by Evrogen (www.evrogen.com).

Cell cultures with constitutive activated *N-Ras* expression were created as previously described [20]. Infected cell cultures were selected for 5-6 days in medium containing 1 μg/mL puromycin (P8833, Sigma) for pBabe and pLKO constructs and 5 μg/mL blasticidin (R210-01, Invitrogen) for pLenti6 constructs. All experiments were performed 5-30 days after vector-mediated gene transfer.

A549 cells were incubated in medium containing 10 μg/ml EGF (E9644, Sigma) and UO126 (V1121, Promega) for 3 days and then western blot analysis and immunofluorescent staining were performed.

### Detection of mRNAs by RT-PCR

Total mRNA was isolated with SV Total RNA Isolation System (Promega) according to the manufacturer's protocols. The following primers were used: β-actin forward 5′-ACAGAGCCTCGCCTTTGC-3′, reverse 5′-GAGGCGTACAGGGATAGCAC-3′; γ-actin forward 5′-CAAAAGGCGGGGTCGCAA-3′, reverse 5′-TGGGGTACTTCAGGGTCAGG-3′; α-tubulin forward 5′-GTTGGTCTGGAATTCTGTCAG-3′, reverse 5′-AAGAAGTCCAAGCTGGAGTTC-3′.

The quantification of mRNA bands was performed using Chemi-Smart 3000 Imaging System (Vilber Lourmat) and TotalLab v.2.01 software.

### Immunoprecipitation and western blot analysis

were performed as previously described [20, 48]. Alexa488-conjugated secondary antibodies were used, band detection was performed using variable mode imager Typhoon9410 (GE Healthcare).

### Antibodies

β-Actin (MCA5775GA, AbD Serotec); γ-actin (MCA5776GA, AbD Serotec); p-p44/42 (T202/Y204) (4370S, Cell Signaling); ERK1/2 (4695S, Cell Signaling); p34-Arc (07-227, EMD Millipore); WAVE2 (3659P, Cell Signaling); Cofilin (D3F9) XP®, cofilin1 (5175P, Cell Signaling); p-Cofilin1 (Ser3) (3313, Cell Signaling); N-ras (sc-519, Santa Cruz); PP1α (S3010, Epitomics); Pan-actin (4968, Cell Signaling); α-tubulin (2144, Cell Signaling).

The following secondary Abs were used: FITC-, TRITC-, AlexaFluor488-, AlexaFluor594-, AlexaFluor647-, Cy5-conjugated goat anti-mouse IgG1, IgG2b, IgG and goat anti-rabbit IgG (Southern Biotechnology, Associates Inc., Birmingham, AL; Jackson; Life technologies).

### Immunofluorescent and confocal laser scanning microscopy

Cells on cover slips were fixed in 1% PFA in pre-warmed DMEM/Hepes for 15 min and treated for 5 min with MeOH at −20°C. Cells were incubated with primary and secondary antibodies. DAPI (Life technologies) was applied for nuclear staining.

Confocal images were acquired using scanning laser confocal microscope with 405 nm 5mW, 488nm 10mW, 555nm 10 mW, 639 nm 5 mW lasers (LSM700, Zeiss, Oberkochen, Germany) equipped with oil immersion objective “Plan-Apochromat” 63x /1.4 (Zeiss) and image software (Zen 2011, Carl Zeiss MicroImaging GHBH, Jena, Germany). Single optical sections were scanned with ~1μm thickness. For serial optical sections stacks with Z-step of 0.2μm were collected.

### Cell cultures growth rate

5×10^4^ А549, 2,5×10^4^ НСТ116 and 2×10^4^ HaCaT cells were seeded into 6-well plates and cell count was performed each two days using the hemocytometer (three wells per time point). The measurement proceeded till monolayer formation.

### Boyden chamber cell migration assay

was performed using transwell Matrigel-coated chambers with 8-μm pore-size membranes (BD Biosciences) according to manufacturer instructions with 5×10^4^ A549, 2,5×10^4^ HCT116 and 2×10^4^ HaCaT cells. The migration activity was quantified by blind counting of the migrated cells of at least 10 fields per chamber.

### Nude mice assay

Nude mice were inoculated with 10^6^ cells as previously described [49-51]. Tumor sizes were measured every 3 days and their volumes were calculated as (width2) × (length) × 0.5. After 3 weeks of observation, explanted tumors were isolated and analyzed. The animal experimental protocols were approved by the Committee for Ethics of Animal Experimentation and the experiments were conducted in accordance with the Guidelines for Animal Experiments in Russian Blokhin Cancer Research Center.

### Patients and tissue material

CRC samples and paired non-cancerous tissues were obtained from the patients of Russian Blokhin Cancer Research Center undergone radical surgery for colorectal cancer and lung cancer. Informed consent has been obtained from patients and the project was approved by the institutional review board. All patients had a definite pathological diagnosis (adenocarcinomas G2 grade) and did not receive chemotherapy or radiotherapy before surgery. Formol-fixed, paraffin-embedded samples of human tissues were obtained from surgical material and paraffin archive blocks of the Division of Clinical Pathology, Russian Blokhin Cancer Research Center. Animal tissue samples were rinsed in ice-cold PBS, fixed for 24 hrs in 4 % formaldehyde and placed in 70 % EtOH until embedded in paraffin. The histological sections (5 μm) were deparafinised in о-xylol twice for 5 min and then three times for 5 min in 96 % EtOH before immunofluorescent staining.

### Immunofluorescent staining of histological sections

Antigen retrieval was achieved by heating at 95°C in target retrieval solution (pH = 6.0) (S1699, Dako) for 40 min. The sections were incubated with primary antibodies at room temperature for 1 hour, followed by incubations with FITC-/TRITC-conjugated secondary antibodies for 30 min at room temperature. Quantitative analysis for the intensity of immunoflurescence was performed using ImageJ software (http://rsbweb.nih.gov) by measuring the specific color intensity. For each sample at least 5 sections were examined, in each section at least 15 fields of vision were analyzed.

### *In situ* proximity ligation assay (PLA)

PLA was conducted according to manufacturer's instructions (Sigma-Aldrich). In brief, PFA/methanol-fixed cells were incubated with pairs of primary antibodies, washed and incubated with secondary antibodies conjugated with oligonucleotides (PLA probe MINUS and PLA probe PLUS). A proximity-dependent ligation and amplification of DNA reporter molecule followed. The resulting rolling circle products (RCPs) were visualized by fluorescence microscopy upon hybridization with a complementary fluorescence-labeled oligonucleotide probe.

### Statistical analysis

Statistical analysis was done with unpaired Student's t tests, and data are expressed as SD as indicated in figure legends. P values ≤ 0.05 were considered to be significant. All the experiments were performed for at least three times.

## SUPPLEMENTARY MATERIALS FIGURES


