# Supplementary material for: Tumor promotion by γ and suppression by β non-muscle actin isoforms
Source: Oncotarget. 2015 May 4;6(16):14556–71. doi: 10.18632/oncotarget.3989 (PMC4546487; doi:10.18632/oncotarget.3989)
Supplement: Supplementary file 1 [file oncotarget-06-14556-s001.pdf]

Tumor promotion by γ and suppression by β non-muscle actin isoforms

Supplementary Material

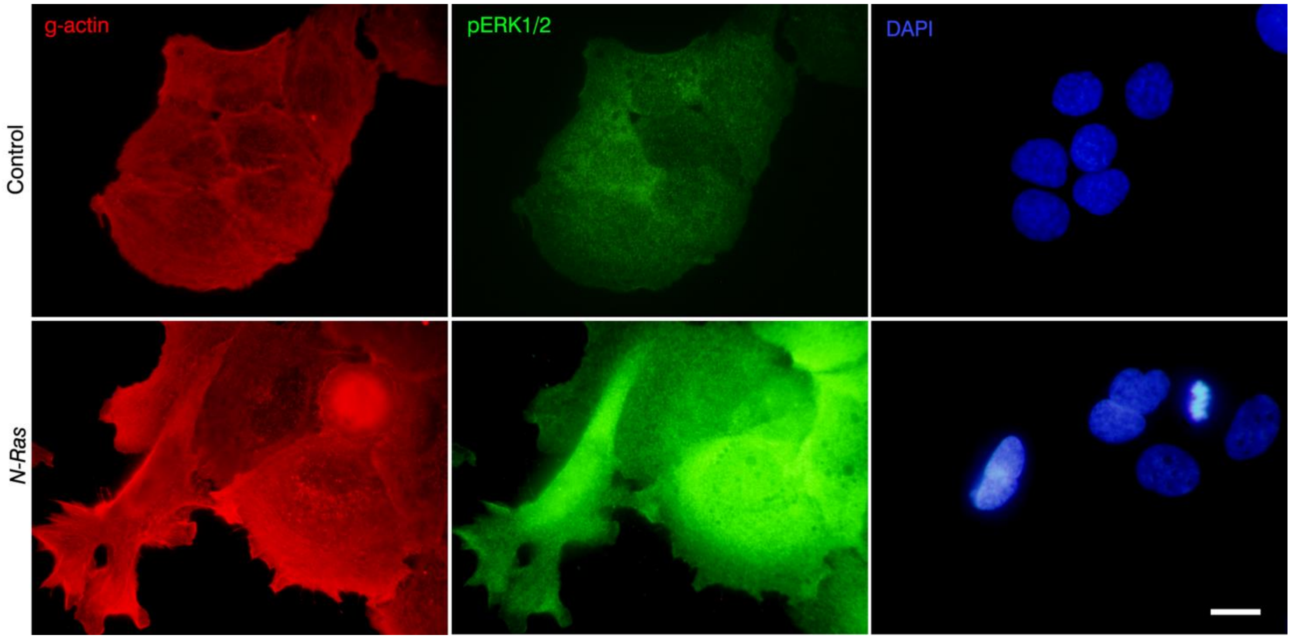

Supplementary Figure 1

LSM of HaCaT cells (upper panel) and with *N-RasD*^{13} expression (lower panel) with γ-actin (red), pERK1/2 (green) immunofluorescent staining. Scale bar represents 10 µm.

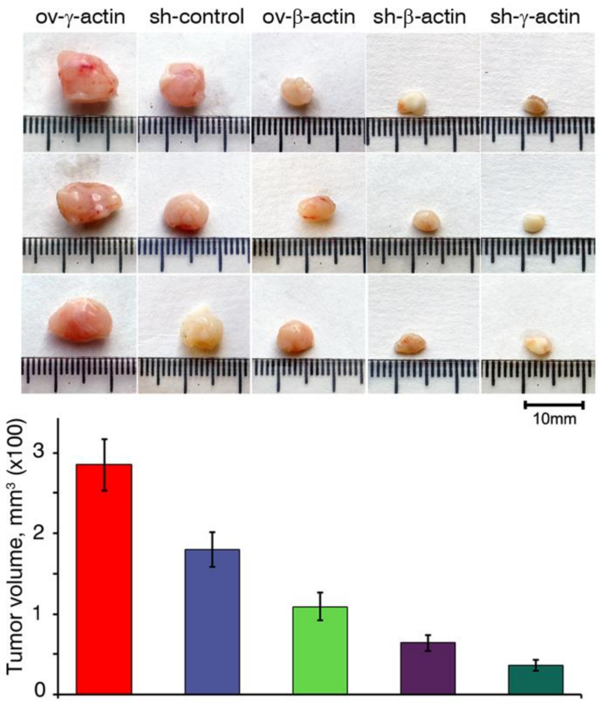

Supplementary Figure 2

Photographs of xenographts of HCT116 cells with exogenous expression of β- or γ-actins and corresponding shRNAs 21 days after subcutaneous implantation. Graphs represent volumes of 10 tumors per each group (Mean ± SD).

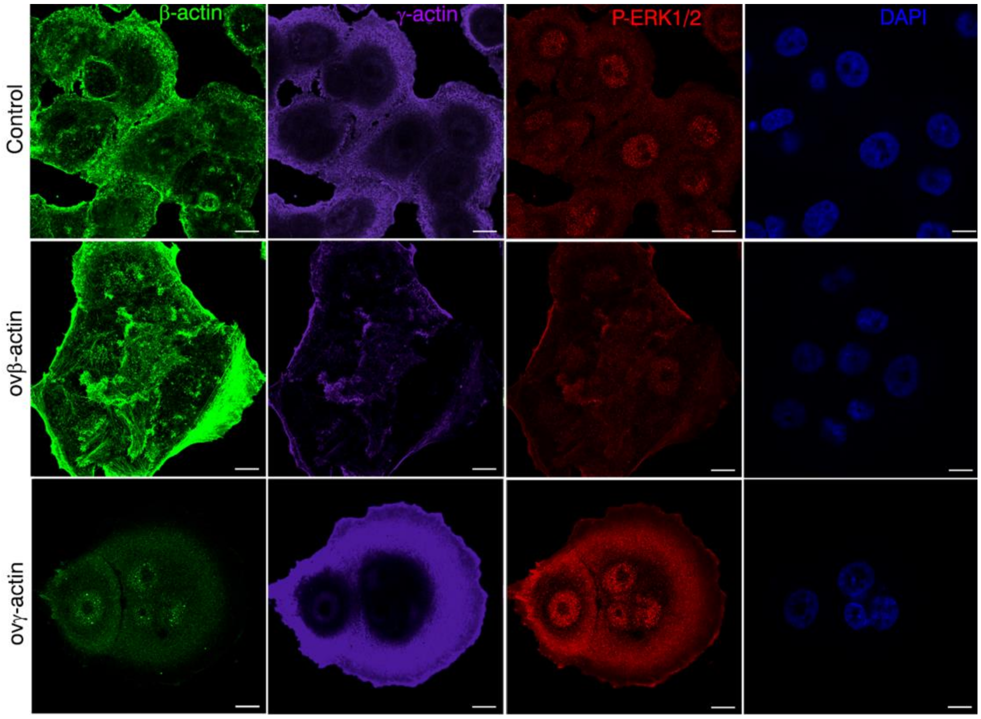

Supplementary Figure 3

LSM of A549 cells with β- or γ-actins overexpression with β-actin (green), γ-actin (purple) or pERK1/2 (red) immunofluorescent staining. Scale bars represent 10 µm.

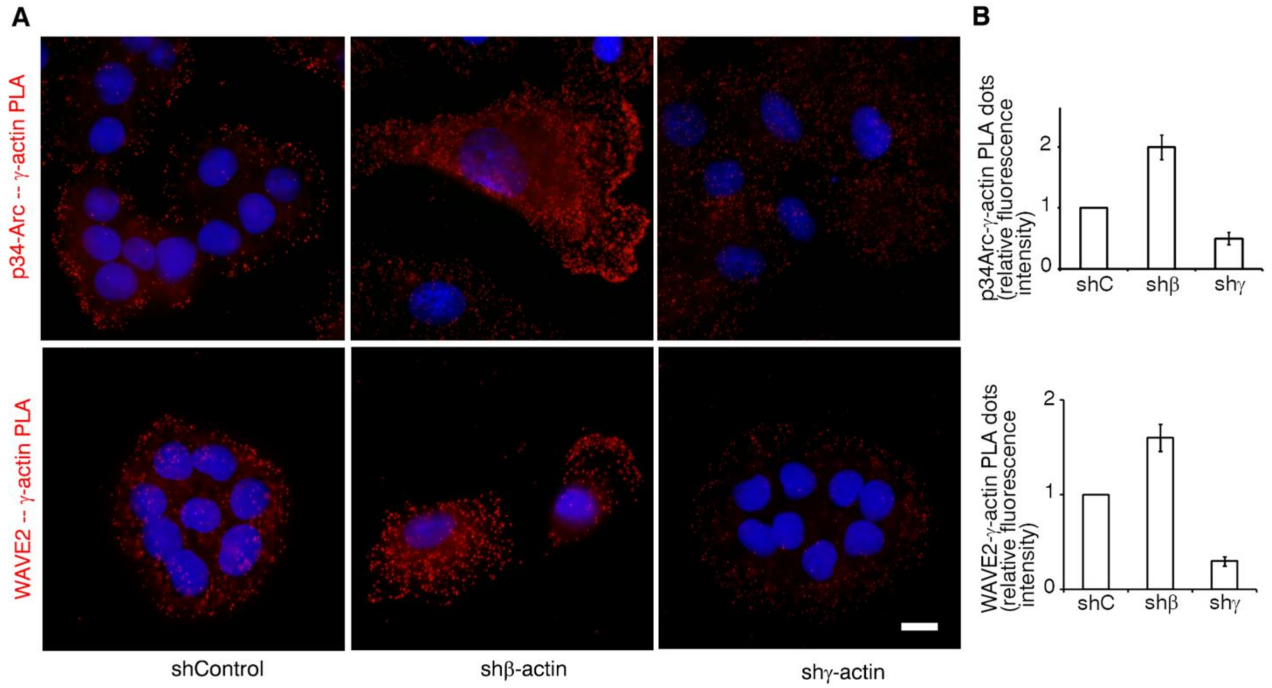

Supplementary Figure 4

p34-Arc/γ-actin (upper panel) or WAVE2/γ-actin (lower panel) PLA analysis of A549 cells with down-regulated β- or γ-actins. Scale bar represents 10 µm. Graphs represent relative fluorescence intensity (Mean ± SD).

A. Fluorescence microscopy of pERK1/2−γ-actin PLA dots.

B. Comparative quantification of pERK1/2−γ-actin PLA dots.

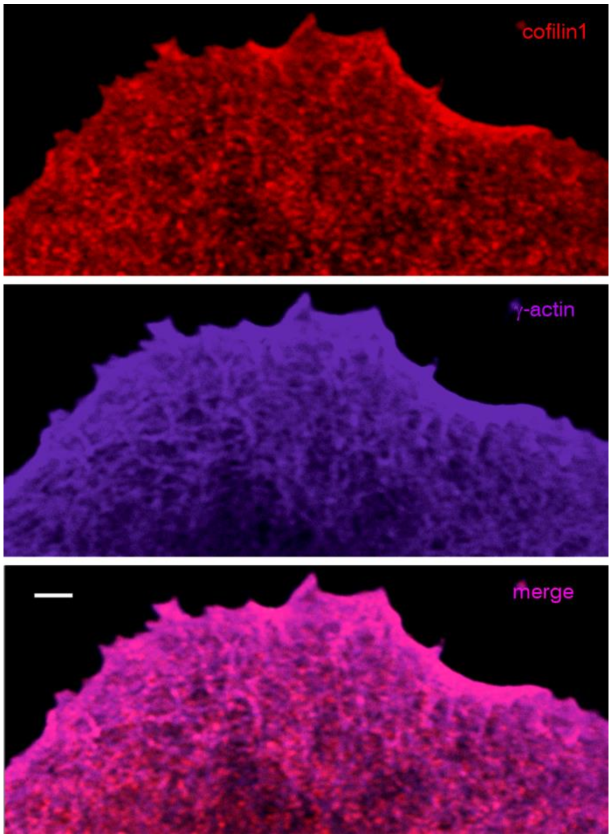

Supplementary Figure 5

LSM of A549 cell with down-regulated β-actin:

γ-actin (purple), cofilin1 (red) immunofluorescent staining, merge (rose). Leading edge fragment is shown. Scale bar represents 2 µm.
